# TGF-β1 promotes acinar to ductal metaplasia of human pancreatic acinar cells

**DOI:** 10.1038/srep30904

**Published:** 2016-08-03

**Authors:** Jun Liu, Naoki Akanuma, Chengyang Liu, Ali Naji, Glenn A. Halff, William K. Washburn, Luzhe Sun, Pei Wang

**Affiliations:** 1 Departments of Cellular and Structural Biology, University of Texas Heath Science Center at San Antonio, 7703 Floyd Curl Drive, San Antonio, TX 78229, USA; 2 Surgery Department, University of Pennsylvania School of Medicine, Philadelphia, PA 19104, USA; 3 Transplant Centre, University of Texas Health Science Center at San Antonio, San Antonio, TX 78229, USA

## Abstract

Animal studies suggest that pancreatitis-induced acinar-to-ductal metaplasia (ADM) is a key event for pancreatic ductal adenocarcinoma (PDAC) initiation. However, there has not been an adequate system to explore the mechanisms of human ADM induction. We have developed a flow cytometry-based, high resolution lineage tracing method and 3D culture system to analyse ADM in human cells. In this system, well-known mouse ADM inducers did not promote ADM in human cells. In contrast, TGF-β1 efficiently converted human acinar cells to duct-like cells (AD) in a SMAD-dependent manner, highlighting fundamental differences between the species. Functionally, AD cells gained transient proliferative capacity. Furthermore, oncogenic KRAS did not induce acinar cell proliferation, but did sustain the proliferation of AD cells, suggesting that oncogenic KRAS requires ADM-associated-changes to promote PDAC initiation. This ADM model provides a novel platform to explore the mechanisms involved in the development of human pancreatic diseases.

Pancreatic ductal adenocarcinoma (PDAC) is among the most deadly human malignancies. Oncogenic KRAS mutation represents the most frequent and earliest genetic alteration in PDAC patients, highlighting its role as a driver of PDAC. However, some healthy individuals carry somatic oncogenic KRAS mutations in the pancreas for years without developing PDAC, suggesting that additional events are required for oncogenic KRAS to initiate PDAC. Among two major types of epithelial (acinar and ductal) cells in the adult exocrine pancreas, ductal cells traditionally were thought to be the cell of origin of PDAC, based on histologic appearance. However, accumulating evidence emphasizes the importance of acinar plasticity in PDAC tumourigenesis^1^,^2^,^3^,^4^,^5^,^6^. Lineage tracing experiments in mouse PDAC models demonstrated that PanIN lesions are mainly derived from acinar cells undergoing acinar to ductal metaplasia (ADM), an event usually induced by pancreatitis^1^,^7^,^8^, suggesting that ADM might be an early event that promotes KRAS-driven PDAC tumourigenesis^1^,^9^. Supporting this view, pancreatitis is the biggest risk factor for PDAC in humans^10^, and experimental pancreatitis is also required for KRAS-driven PDAC initiation in adult mice^11^,^12^.

Recently, mechanistic studies of ADM in murine pancreatic acinar cells have continued to evolve. TGF-α, a member of the epidermal growth factor (EGF) family, and oncogenic KRAS are capable of driving ADM in mice, possibly via activation of the MEK/ERK pathway^5^,^13^,^14^,^15^,^16^,^17^. More recently, activated macrophages have been demonstrated to secrete cytokines that can promote ADM of mouse acinar cells^14^. This inductive effect is largely mediated by TNFα (tumour necrosis factor α), RANTES (Regulated upon activation normal T cell expressed) and some unknown soluble factors secreted by activated macrophages, which may play essential roles in mediating inflammation-induced ADM in experimental animal models. However, it is unclear whether human and mouse cells induce ADM via the same mechanisms. The most recently published attempts to model PDAC by culturing human pancreas organoids yielded only ductal cells, not acinar cells^18^. Houbracken *et al*. have showed human primary acinar cells undergoing ADM *in vitro* using cell clusters^19^, but this method is not compatible with further functional studies. Currently, there has not been an adequate system to explore the mechanism for ADM induction in humans and the contribution of ADM to human PDAC tumourigenesis.

To investigate the ADM process in human cells, we developed a system to identify, separate, and genetically manipulate human primary pancreatic acinar and ductal cells. With this system, we showed that human cells need different signals than do mouse cells to induce ADM, and demonstrated that cells undergoing ADM (AD cells) can form spheres in 3D culture, reflecting a transient activation of proliferation. Moreover, oncogenic KRAS expression did not induce sphere formation ability in human acinar cells, but permitted expansion of AD cell-derived spheres during prolonged culture. Our study not only highlights the different signals required by human and mouse cells to induce ADM, but this new system also provide a platform to investigate the initiation of PDAC tumourigenesis in human cells.

## Results

### Characterization of acinar and ductal populations in primary human exocrine pancreatic tissues

**Figure 1:**
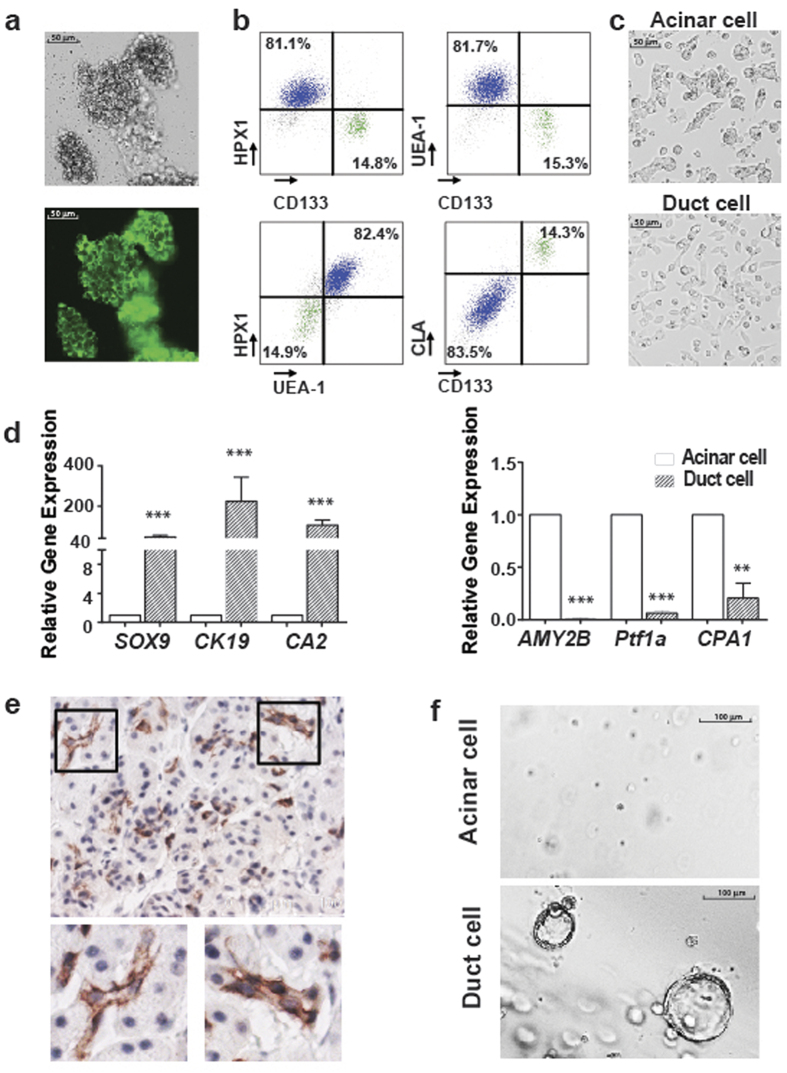
Characterization of human primary pancreatic acinar and ductal cells. (**a**) A significant proportion of cells in the acini showed strong UEA-1 staining on the cell membrane. (**b**) Two major distinct populations, characterized by HPX1^+^UEA-1^high^CLA^−^CD133^−^ acinar cells (blue) and HPX1^−^UEA-1^low^CLA^+^CD133^+^ ductal cells (green), were identified in fresh tissues (n = 16). (**c**) Acinar cells displayed a cobblestone shape and aggregated to form small clusters. Ductal cells spread out to assume a spindle-like shape. (**d**) Acinar cells expressed high levels of acinar markers *AMY2B*, *PTF1a*, *CPA* and ductal cells expressed high levels of ductal markers *CA2*, *CK19*, *SOX9* (n = 3). Data represent means ± s.e.m. from three independent experiments **P < 0.01, ***P < 0.001. (**e**) CLA is specifically expressed in ductal cells. (**f**) Ductal cells, but not acinar cells, could form ring-like spheres in the 3D-embedded culture system.

A few studies have reported the ADM in human cells but they did not isolate the viable primary human pancreatic acinar and ductal cells for subsequent functional studies. To distinguish different cell types in the normal human islet-depleted pancreatic exocrine tissue fraction, we used several cell surface markers to analyse the cells by flow cytometry (Supplementary Fig. 1a). Less than 2% of the cells derived from these tissues were CD45^+^ hematopoietic cells or CD31^+^ endothelial cells (Supplementary Fig. 1b). The majority (>98%) of cells were positive for the epithelial marker CD326, also called EpCAM (Epithelial cell adhesion molecule), consistent with the epithelial origin of the exocrine pancreas (Supplementary Fig. 1c). Ulex europaeus agglutinin 1 (UEA-1) has been used to label both mouse and human pancreatic acinar cells^19^,^20^. As shown in Fig. 1a, the acinar clusters can be stained with FITC-conjugated UEA-1. To confirm whether UEA-1 can bind specifically to acinar cells, exocrine pancreatic cells labelled with FITC-UEA-1 were further stained with the ductal cell marker CD133^21^ and the acinar cell marker HPX1^22^, and analysed by flow cytometry. As expected, HPX1 and CD133 staining were mutually exclusive in these tissues, suggesting that they specifically stained acinar and ductal cells, respectively. HPX1 stained UEA-1^high^ cells, while CD133 stained UEA-1^low^ cells (Fig. 1b). Thus, acinar and ductal populations were readily recognized, respectively, by UEA-1^high^CD133^−^ and UEA-1^low^CD133^+^ surface staining patterns. Using these markers to distinguish acinar and ductal cells, we found that the frequencies of acinar cells varied from 44% to 82% (n = 16) in the samples analysed, possibly due to variations in tissue sources or preparation.

We found that CD146, SSEA-4, and CLA (Cutaneous lymphocyte-associated antigen) were preferentially expressed by ductal cells (Fig. 1b; Supplementary Fig 1b–e). Further validation with CD133 and CLA staining confirmed that CLA was highly specific for ductal cells (Fig. 1b). We further examined the expression of CLA in human pancreatic tissue sections by immunohistochemistry. As shown in Fig. 1e, CLA specifically stained ductal structures in normal human pancreatic tissues, suggesting that CLA is a novel surface marker for human pancreatic ductal cells. Using a panel of cell surface markers, we define acinar and ductal cells as UEA-1^high^CLA^−^CD133^−^ and UEA-1^low^CLA^+^CD133^+^, respectively.

To further confirm the cellular origins of these two populations, we sorted acinar and ductal cells from exocrine pancreatic tissue and cultured them in serum-free media. One day after culture, they assumed different morphological features: acinar cells were bigger and epithelial-like, while ductal cells were long and spindle shaped (Fig. 1c), consistent with the distinct lineage origins of these cells. Furthermore, we evaluated the RNA expression of a set of acinar and ductal markers in sorted cells by qRT-PCR. Indeed, UEA-1^low^CLA^+^CD133^+^ cells expressed high levels of ductal markers, such as *CA2*, *CK19* and *Sox9*, while UEA-1^high^CLA^−^CD133^−^ cells preferentially expressed acinar markers including *AMY2B* (Pancreatic Amylase-α2B), *CPA* and *PTF1a* (Fig. 1d, n = 3, P <  0.01), further supporting the identity of these two populations as ductal cells and acinar cells, respectively.

Ductal cells have previously been shown to form spheres *in vitro*^21^. We investigated whether acinar cells have the same property. The acinar and ductal cells were sorted and placed in sphere formation assays. Consistent with the previous report, the ductal cells could reproducibly give rise to ring-like spheres in Matrigel 3D culture, whereas UEA-1^high^CLA^−^CD133^−^ acinar cells did not form spheres (Fig. 1f). Taken together, these results demonstrated that of UEA-1, CLA, and CD133 surface markers can be used for prospective identification and separation of viable human primary pancreatic acinar and ductal cells.

### Induction of ADM by TGF-β1 in human acinar cells

Several cytokines have been shown to induce mouse acinar cells to undergo ADM^14^. Using the Matrigel-embedded 3D culture system, we screen the ADM inducers for human cells. We screen a panel of inflammation-or PDAC-associated cytokines and growth factors, including TGF-α, TGF-β1, EGF, SHH, VEGF, TNF-α, RANTES, IL-1β, IL-6, IL-8, and CXCL12. Among these tested cytokines, only TGF-β1 promoted the conversion of acinar cells to CD133^+^ ductal-like cells in 3D culture (Supplementary Fig. 2).

**Figure 2:**
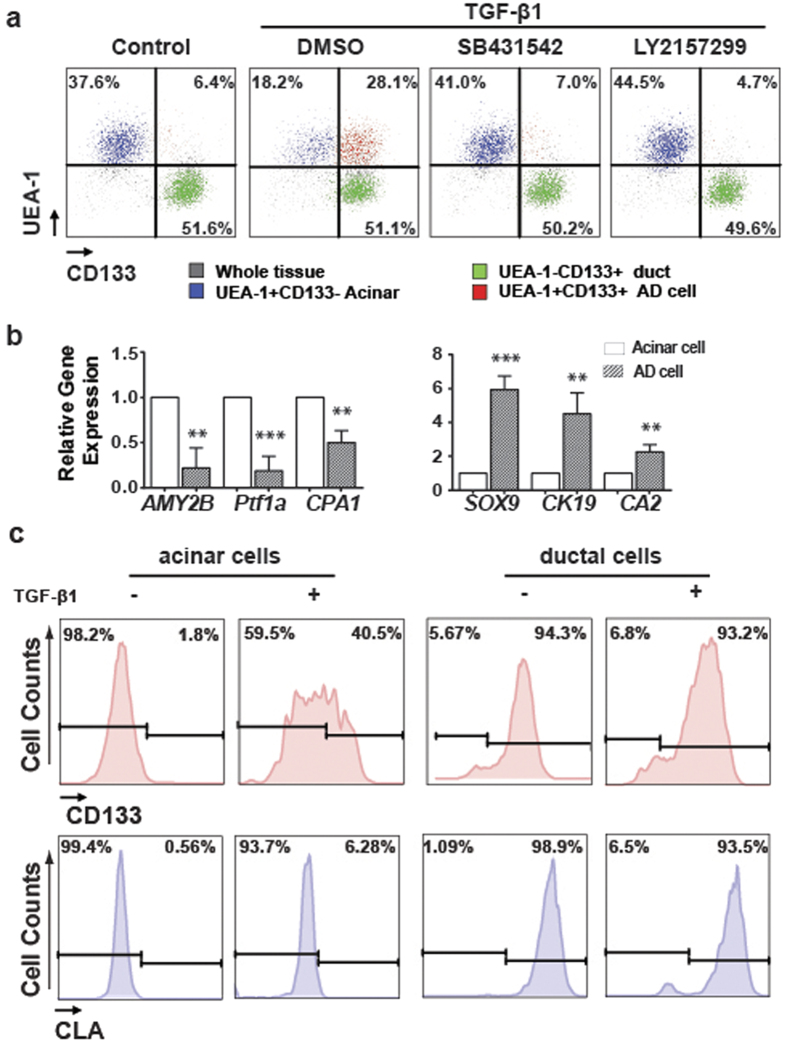
TGF-β1 directly promotes ADM of human primary pancreatic acinar cells. **(a**) Compared with untreated control culture, TGF-β1 treatment for 3 days strongly induced the conversion of acinar cells (blue) to ADcells (red). Ductal cells (green) showed no significant change after TGF-β1 treatment. SB431542 and LY2157299 (TGF-β receptor1 inhibitors) treatment abolished conversion induced by TGF-β1 (n = 6). (**b**) Comparison of the expression of acinar and ductal markers in sorted acinar cells and AD cells by qRT-PCR assay. AD cells sorted from TGF-β1-treated tissues expressed significantly higher levels of ductal-specific genes and lower levels of acinar-specific genes than did acinar cells sorted from untreated tissues (n = 3). Data represent means ± s.e.m. from three independent experiments. **P < 0.01 ***P < 0.001. (**c**) TGF-β1 treatment directly induced the conversion of sorted acinar cells to AD cells. However, TGF-β1 treatment showed no significant effect on sorted ductal cells.

TGF-β1 is a growth factor that plays important roles in mediating chronic inflammation-associated tissue injuries in multiple organs^23^,^24^,^25^. The up-regulation of TGF-β1 has also been well-documented in human pancreatitis^26^,^27^,^28^. We therefore focused on whether TGF-β1 played a role in inducing ADM. We treated 3D cultured exocrine pancreatic tissue with 5 ng/ml TGF-β1 and analysed cell surface marker expression by flow cytometry. We found that TGF-β1 treatment strongly induced the generation of CD133^+^ ductal-like cells from acinar cells (Fig. 2a). After only 3 days of treatment, a significant proportion of acinar cells became UEA-1^high^CLA^−^CD133^+^ ductal-like cells. We observed that the induction efficiency ranged from 28.1% to 87.6% (n = 8) among the tissues tested. We then tested whether TGF-β1 induced ADM through TGF-β receptor 1 (TGFBR1). We examined the effects of SB431542 and Ly2157299, two kinase inhibitors for TGFBR1, on TGF-β1-induced ADM in 3D culture. The primary tissues were pre-labelled with UEA-1-FITC and treated with TGF-β1 in the absence or presence of SB431542 (1 uM) and Ly2157299 (1 uM) for 3 days in 3D culture. Strikingly, SB431542 and Ly2157299 almost completely prevented the TGF-β1-induced transition of acinar cells to CD133^+^ ductal-like cells (Fig. 2a). To examine ADM-associated alterations, we compared the expression of acinar and ductal markers from acinar cells sorted from the control group and UEA-1^high^CLA^–^CD133^+^ cells sorted from TGF-β1-treated tissues (Fig. 2b). UEA-1^high^CLA^−^CD133^+^ cells showed significant down-regulation of acinar markers (*AMY2B* and *PTF1*) and up-regulation of ductal markers (*CK19* and *SOX9*) (n = 3, P < 0.01). These data suggested that TGF-β1 promoted ADM in primary human pancreatic cells and we defined UEA-1^high^CLA^−^CD133^+^ as AD cells.

To directly test whether TGF-β1 could convert acinar cells into AD cells, we sorted acinar cells and ductal cells from primary tissues and cultured them. Three days after culture, acinar cells remained to be CD133 negative. However, a significant portion of acinar cells gained CD133 expression after TGF-β1 treatment (Fig. 2c). However, ductal cells retained their CLA^+^ phenotype, regardless of TGF-β1 treatment (Fig. 2c). Thus, these data clearly demonstrated that TGF-β1 signalling pathway activation directly promoted the ADM of primary human pancreatic acinar cells.

### The signalling pathways mediating ADM

**Figure 3:**
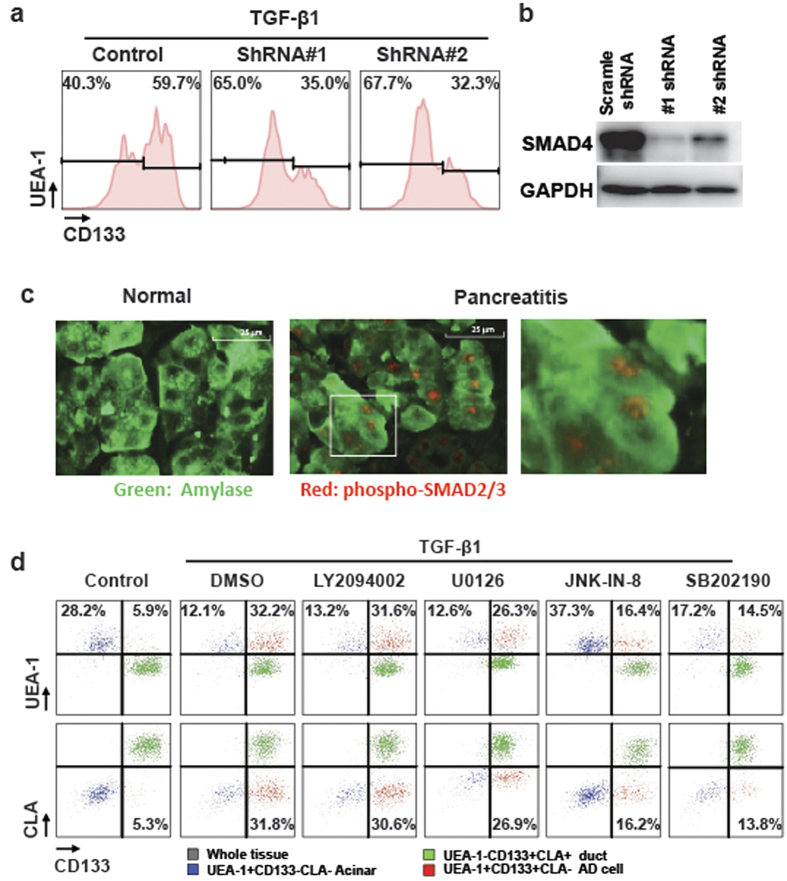
Signalling pathways involved in TGF-β1-induced ADM of human primary pancreatic acinar cells. **(a**) Compared with control lentiviral shRNA transduction, SMAD4 lentiviral shRNA transduction significantly inhibited the CD133 up-regulation induced by TGF-β1 in sorted acinar cells (n = 3). (**b**) Two different lentiviral shRNAs efficiently knockdown SMAD4 protein in primary human exocrine pancreatic tissues. (**c**) Compared with the healthy normal pancreas, the p-SMAD3 level increased in acinar cells exposed to an inflammatory environment *in vivo*. (**d**) Treatment with Ly294002 (PI3K inhibitor) had no significant effect on the TGF-β1-induced conversion of acinar cells to AD cells. However, treatment with U0126 (MER inhibitor), SB202190 (P38 inhibitor), and JNK-IN-8 (JNK inhibitor) partially inhibited this conversion (n = 3).

TGF-β1 is known to activate both the canonical SMAD pathway and non-canonical pathways. To examine the role of SMAD pathway activation in ADM induced by TGF-β1, we sorted primary acinar cells and transduced them with 2 different Lentiviral shRNAs against SMAD4, then placed the cells in culture, followed by TGF-β1 treatment (n = 3). Flow cytometry showed that SMAD4 knockdown significantly inhibited the TGF-β1-induced transition of acinar cells to AD (Fig. 3a). We found that SMAD4 was efficiently knocked-down by both shRNAs (Fig. 3b). This result suggests that canonical SMAD signalling pathway activation is required for TGF-β1 to induce ADM of primary human pancreatic acinar cells. We used a tissue array to examine the p-SMAD3 expression levels in 5 normal tissues and 16 cancer-adjacent tissues by immunofluorescence. As shown in Fig. 3c, the acinar cells were negative for p-SMAD3 in 4 of 5 normal tissues tested, suggesting that TGF-β/SMAD signalling is not activated under normal physiological conditions. By contrast, in 12 of 16 cancer-adjacent tissues exposed to inflammatory insults, acinar cells showed significant elevation of p-SMAD3 expression. These data indicated the prevalence of SMAD signalling activation in human pancreatic acinar cells in inflammatory environments.

TGF-β1 can also activate non-canonical pathways, including PI3K, ERK, JNK, and P38^29^. Therefore, we evaluated the potential contributions of these non-SMAD pathways to TGF-β1-induced ADM in 3D culture. The primary tissues were pre-stained with UEA-1-FITC and embedded into Matrigel, then cultured with TGF-β1 in the presence of LY294 002 (Pan PI3K inhibitor), U0126 (Pan MEK inhibitor), JNK-IN-8 (Pan JNK inhibitor), or SB202190 (Pan p38 inhibitor) for 3 days. Blocking the PI3K pathway showed no significant effect on TGF-β1-induced ADM. However, blocking ERK, JNK, or P38 partially inhibited TGF-β1-induced conversion of acinar cells to AD cells in 3D culture (Fig. 3c). This result suggests that the activation of these pathways may also contribute to TGF-β-induced ADM of human primary pancreatic acinar cells.

### Proliferation capacity of AD cells

**Figure 4:**
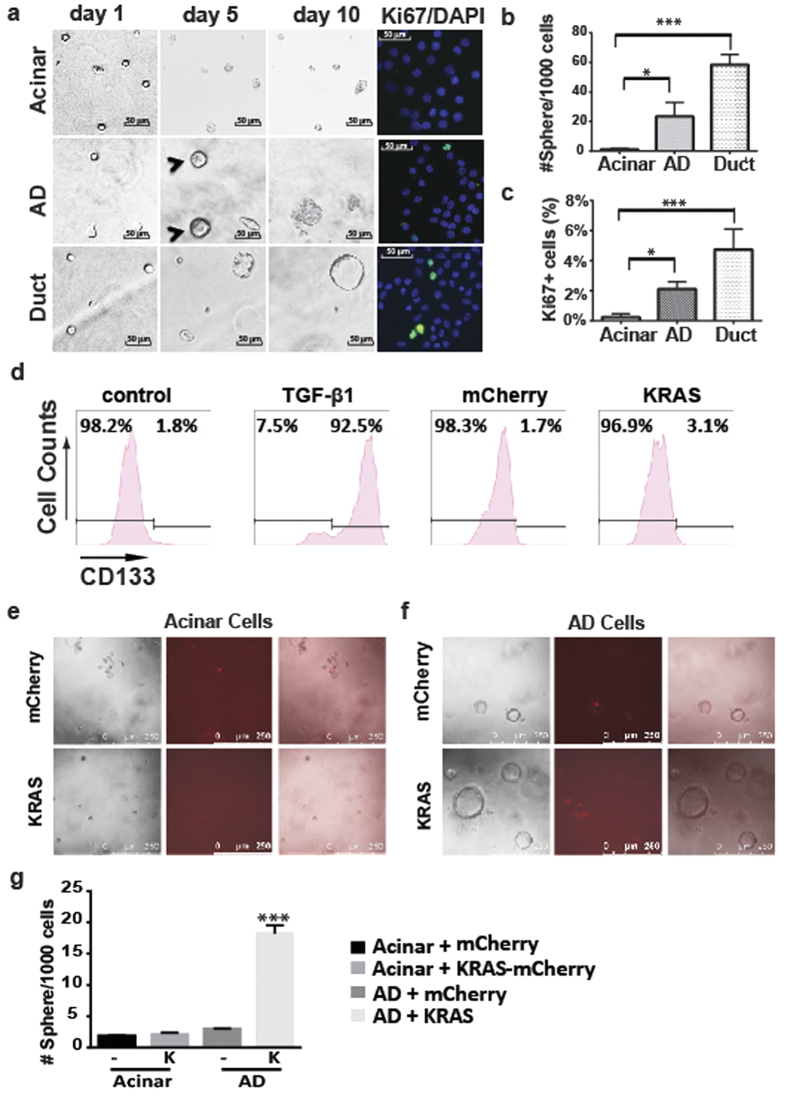
Oncogenic KRAS promotes AD cells toward further proliferation. (**a**) In the 3D-embeded system, while ductal cells proliferated continuously to form large spheres, and acinar cells could not make spheres, AD cells acquired the ability to proliferate transiently to form small spheres (arrowhead). (**b**,**c**) Quantification of sphere formation and Ki67 positive cells for sorted acinar, ductal and AD cells (n = 3). Data represent means ± s.e.m. from three independent experiments *P < 0.05, ***P < 0.001. (**d**) Expression of oncogenic KRAS did not induce ADM in primary human acinar cells. (**e**,**f**) Oncogenic KRAS enhanced the sphere formation capacity of AD cells but not of acinar cells. (**g**) Quantification of spheres formation assay (n = 3). Data represent means ± s.e.m. from three independent experiments ***P < 0.001.

Ductal cells are proliferative and can form ring-like spheres, while acinar cells have neither of these properties. To test whether AD cells remain like acinar cells or form spheres like ductal cells, we sorted AD cells and acinar cells from the tissues treated with TGF-β1 in 3D culture. Similar to fresh primary acinar cells, acinar cells did not form spheres. In contrast, AD cells could form small spheres by seven days, although most of these spheres began to die after 10 days in culture, suggesting a transient activation of proliferation in AD cells (Fig. 4a,b). To further confirm the activation of proliferation in AD cells, we performed Ki67 staining. Consistent with the sphere formation assay, we found some AD cells expressed Ki67, while the cultured acinar cells did not (Fig. 4a,c, n = 3, P < 0.05).

Oncogenic KRAS induces ADM in mouse acinar cells^5^. We tested whether oncogenic KRAS can convert human acinar cells to AD cells by transducing sorted acinar cells with oncogenic KRAS (n = 3). As shown in Fig. 4d, although TGF-β1 treatment induced ADM in sorted acinar cells, expression of oncogenic KRAS could not convert acinar cells to CD133^+^ AD cells. Finally, we tested the effect of oncogenic KRAS on the sphere formation capacity of human acinar cells. We treated sorted acinar cells with TGF-β1 containing medium to induce ADM. In the control group, TGFBR1 inhibitor SB431542 was added to prevent ADM induction. Five days after culture, we transduced the cells with lentiviral particles expressing KRAS-mCherry or mCherry alone and assessed the cells’ sphere formation in 3D culture. During 3 weeks in culture, we found that the cells cultured in control group did not form spheres with either KRAS-mCherry or mCherry (Fig. 4e). In contrast, the cells treated with TGF-β1 containing medium and transduced with mCherry virus generated spheres and died off by 3 weeks. the cells treated with TGF-β1 containing medium and transduced with KRAS-mCherry virus formed significantly more and bigger spheres (Fig. 4f,g, n = 3, P < 0.001). Collectively, these data showed that oncogenic KRAS did not induce acinar cells to undergo ADM, but did extend the proliferation of AD cells.

## Discussion

In the current study, we used a panel of cell surface markers, UEA-1, HPX1, CD133, and CLA for analysing primary human exocrine pancreatic tissues by flow cytometry. The acinar cell-specific antibody HPX1^22^ mainly stained UEA-1^high^ cells, while the ductal cell-specific antibody CD133^21^ predominantly stained UEA-1^low^ cells, which is consistent with the previous observation that UEA-1 specifically binds to the acinar cells in human exocrine pancreas^19^. The introduction of flow cytometry improved the resolution of lineage tracing method and allowed us to directly analyse the gene expression of sorted acinar and ductal cells. Indeed, gene expression analysis demonstrated that UEA^high^CD133^−^ and UEA^low^CD133^+^ populations were enriched for expression of acinar markers (*AMY2B*, *CPA1,* and *PTF1a*) or ductal markers (*Sox9*, *Ck19,* and *CA2*), respectively. HPX1 staining was reduced after culture, making it unsuitable for lineage tracing experiments. Although we observed various intensities of UEA-1 staining among different tissue samples, we found when tissue was pre-stained with UEA-1 that the UEA-1 staining remained constant during subsequent culture, suggesting that UEA-1 can be used for lineage tracing of acinar cells.

The previous studies proposed that selective survival of the ductal cells might explain the enrichment of cells with a ductal phenotype in primary human exocrine pancreatic fractions during their culture^30^,^31^. We also found that the frequency of CD133^+^ cells increased significantly during culture. However, the frequency of cells of acinar origin only decreased modestly in our culture conditions. Thus, survival selection alone could not explain the enrichment of CD133^+^ cells in our study. Furthermore, sorted acinar cells can be induced to the AD phenotype, indicating these cells were directly derived from UEA-1 pre-labeled acinar cells. In addition to gaining CD133 expression, these cells also showed up-regulation of ductal markers (*Sox9*, *Ck19*) and down-regulation of acinar markers (*AMY2B*, *PTF1a*) when compared with UEA-1^high^CD133^−^ cells, thus supporting the idea that acinar cells have the potential to convert to ductal-like cells (AD cells).

We identified several new markers preferentially expressed by ductal cells. Among these genes, *CLA* is a highly specific marker for cells of ductal origin. When the acinar cells gained CD133 expression to become ductal-like cells, they remained CLA negative, further demonstrating that AD cells differ from ductal cells. By immunohistochemistry, we found that ductal cells in human pancreas express CLA. To our knowledge, CLA expression in pancreatic tissue has not been described. This highly specific expression pattern might suggest that CLA has unique functions in pancreatic ductal cells and would be of interest for further investigation. Despite the significant up-regulation of ductal markers in AD cells, their expression levels were still much lower than the levels observed in ductal cells (Fig. 2b), indicating that AD cells are different from ductal cells.

The previous lineage tracing study in human tissues did not study the functional changes of ADM cells^19^. Our flow cytometry-based method allowed us to directly study the cells undergoing ADM-associated changes. When cultured in Matrigel with a serum-free culture media, sorted ductal cells continuously proliferated and reproducibly formed large spheres, but acinar cells did not form spheres under the same conditions, which is consistent with previous studies^21^. Interestingly, AD cells could form small spheres after a week, which recapitulated the observation from a mouse model, which showed that metaplastic acinar cells are proliferative^32^. However, most of these spheres could not grow continuously during prolonged culture, suggesting that the proliferation in AD cells was transient, and further supporting the observation that AD cells are intrinsically different from ductal cells. Interestingly, our data also showed that enforced expression of oncogenic KRAS significantly enhanced the sphere formation capacity of cells undergoing ADM but had no obvious effect on acinar cells. It would be interesting to test the hypothesis that transient activation of proliferation in AD cells is an intrinsic mechanism for tissue repair and regeneration, which might be hijacked by oncogenic KRAS to initiate PDAC formation. Nevertheless, the clinical relevance of this *in vitro* model needs to be rigorously tested in pancreatitis and PDAC patients in the future.

Little is known about the difference between the ADM-inducing mechanisms in human and mouse cells. Oncogenic KRAS can induce ADM in murine pancreatic acinar cells, but we found that enforced expression of oncogenic KRAS in primary human pancreatic acinar cells neither induced CD133 expression nor enhanced cell proliferation, demonstrating a difference between human and murine acinar cells. Consistent with this observation, we found that the EGFR ligands EGF and TGF-α, which can induce ADM in mouse cells^13^,^15^, could not induce ADM in primary human acinar cells. In addition, several other factors, such as TNF-α, IL6, and IL1-α, which can induce ADM of murine cells, have no effect on primary human pancreatic acinar cells. The different responses of human and murine acinar cells to these factors might be due to species differences, and highlight the importance of studying human cells.

TGF-β1 is a homodimeric, multifunctional cytokine. TGF-β1 expression increases significantly in both human chronic pancreatitis and experimental pancreatitis in animal models^26^,^27^,^33^,^34^. However, the exact role of TGF-β in the development of pancreatitis-associated ADM remains unclear. One recent study showed that activation of the TGF-β signalling pathway in murine acinar cells limits ADM formation in a cell-autonomous manner^35^. In contrast, we identified TGF-β1 as a potent inducer of ADM in human cells during *in vitro* culture. We showed that TGF-β1 treatment is sufficient to convert isolated acinar cells to AD cells, which supports the cellular plasticity of human acinar cells under *in vitro* culture condition.

We also demonstrated that activation of the SMAD pathway is required for TGF-β1 induced ADM, since knock-down *SMAD4* by 2 independent shRNAs in sorted human acinar cells significantly prevented ADM induced by TGF-β1. *SMAD4* is among the most frequently mutated genes in PDAC patients. However, *SMAD4* inactivating mutations have only been found in high grade pancreatic intraepithelial neoplasia (PanINs)^36^, raising the possibility that SMAD4 signalling is temporally required to induce ADM for the initiation of PDAC. The effect of SMAD4 deletion on KRAS-driven PDAC development has been studied in *Pdx1*^*Cre*^*Kras*^*G12D*^*Smad4*^*lox/lox*^ mouse models in which the genetic alteration occurs in all pancreatic cell types, due to the expression of CRE recombinase at an early developmental stage^37^,^38^. Interestingly, these animals rapidly developed tumours resembling intraductal papillary mucinous neoplasia (IPMN) and MCN^37^, which are more likely to be of ductal origin^39^. In contrast, *Pdx1*^*Cre*^*Kras*^*G12D*^ mice developed PanINs, which are mainly derived from acinar cells. These data indicate SMAD4 deficiency might enhance the susceptibility of ductal cells or alternatively decrease the susceptibility of acinar cells toward KRAS-driven PanINs development. Thus, it would be of interest to re-evaluate dynamic roles of *Smad4* in PDAC development with more elegant animal models which can specially knockout the *Smad4* gene in adult acinar cells or ductal cells. Activation of the MAPK pathways may also contribute to ADM induction, since individual inhibition of the ERK, JNK and P38 pathways by specific small molecule inhibitors partially prevented ADM. Nevertheless, it is unclear whether these pathways are directly induced by TGF-β or by the culture conditions in our system.

In summary, we have developed an ADM model from primary human exocrine tissues to show that TGF-β1 can directly convert acinar cells to ductal-like cells. This model provides the opportunity to directly analyse the gene expression and functional changes that occur when human acinar cells are undergoing ADM. Since this unique model is also compatible with genetic modification techniques, it also has the potential to facilitate engineering of novel human PDAC tumourigenesis models in the future.

## Materials and Methods

### Cell preparation and cell culture

Human islet-depleted cell fractions were obtained from healthy, non-diabetic organ donors deceased due to acute traumatic or anoxic death by the Surgery Department, University of Pennsylvania School of Medicine and by Prodo Laboratories, Inc, and were shipped overnight to our laboratory. Tissues were maintained as previously described^21^. On receipt, the cell fractions were washed with PBS and incubated for 30 minutes with 100 μg/mL fluorescein isothiocyanate (FITC)-conjugated UEA-1 (Sigma-Aldrich, L9006) in CMRL media without serum. For 3D embedded culture, Matrigel (Corning, 354230) was added to tissues suspended in serum free advanced DMEM/F-12 media to a final concentration of 50%. The mixture was placed around the bottom rim of the wells. After solidification at 37 °C for 60 min, wells were overlaid with serum free advanced DMEM/F-12 media supplemented with 10 μM Rock inhibitor Y27632. To induce ADM, tissues were treated with growth factors and cytokines from the start of culture. TGF-β1 was used at the final concentration of 5 ng/ml. IL-1β, IL-8, TNF-α (Genescript), RANTES, TGF-α (Pepro Tech), CXCL12, IL-6, SHH, VEGF (R&D system) and EGF (Sigma) were used at a final concentration of 100 ng/ml. To inhibit the activation of signalling pathways, tissues were pre-treated with inhibitors for 1 hour, followed by addition of TGF-β1. All inhibitors were purchased from Selleckchem. SB431542 (S1067) and LY2157299 (S2230) were used at the final concentration of 1 μM. U0126 (S1102), SB202190 (S1077), and JNK-IN-8 (S4901) were used at the concentration of 2.5 μM. The media was changed daily for all experiments.

### Flow cytometry analysis and cell sorting

Flow cytometry assays were performed with BD LSRII (BD Biosciences) and cell sorting experiments were performed with BD Accuri (BD Biosciences). For uncultured tissues or 2D cultures, the cells were washed with PBS and trypsinised with 0.05% Trypsin-EDTA solution (Life Technologies) for 5 min. Cells were washed with FACS buffer (10 mM EGTA, 2% FBS in PBS) and collected by centrifugation. The pellet was then digested in 1 U/ml dispase solution (Life Technologies) containing 0.1 mg/ml DNaseI in PBS at 37 °C for 5 min. Cells were washed and suspended in FACS buffer for further subsequent antibody staining for flow cytometry. The data were analysed with FlowJo software (FLOWJO, LLC, Ashland, OR).

For 3D cultures, Matrigel was first digested with 1 U/ml dispase solution to release the tissues. The tissues were then prepared as described for uncultured tissues or 2D culture. For analysis of the BD Lyoplate Human Cell Surface Marker Screening Panel (BD Biosciences), we followed the manufacture’s protocol. For cell sorting, trypsin digestion was stopped by adding serum. Dissociated cells were passed through a 40 μm cell strainer and stained with biotin-conjugated CD133 antibodies (clone AC133 and 293C3, Miltenyi Biotech) for 15 min at 4 °C. After washing, cells were stained with APC-conjugated Streptavidin, Pacific blue-conjugated CLA, and 7AAD (BioLegend, San Diego, CA) for 15 min at 4 °C. Cell pellets were collected by centrifugation and washed with PBS after each staining step. The cells were sorted using a FACS Aria II (BD Biosciences) and collected in 100% FBS. After sorting, cells were washed with serum-free Advanced DMEM/F-12 media (Life Technologies), and resuspended in ice-cold Advanced DMEM/F-12 media.

For sphere formation, 30 μl of growth factor-reduced Matrigel was then added to 30 μl cell suspension (8,000 cells), and the mixture was placed around the bottom rim of each well in 48-well plates. After solidification at 37 °C for 60 min, each well was overlaid with 300 μl of Advanced DMEM/F-12 media supplemented with recombinant human (rh) EGF (50 ng/ml, Sigma), rhR-spondin I (500 ng/ml, R&D systems), rhFGF10 (50 ng/ml, R&D systems), recombinant mouse Noggin (100 ng/ml, R&D systems), and 10 mM Nicotinamide. Media was changed twice per week. For 2D culture, 96-well plates were coated with 5% Matrigel. The sorted cells were suspended in Advanced DMEM/F-12 media supplemented with 5% serum and 10 μM Y27632 for 24 hours. Thereafter, cells were cultured in serum-free Advanced DMEM/F-12 media supplemented with 10 μM Y27632. For 3D-on-top culture, 96-well plates were coated with 50% Matrigel to form a thick layer on which cells were seeded.

### Immunohistochemistry

The tissue sections were deparaffinised and incubated in Antigen Unmasking Solution (Vector laboratories, H-3300) at 95 °C for 12 min. Then the sections were treated with methanol containing 3% hydrogen peroxide for 15 min at room temperature to reduce endogenous peroxidase activity and were blocked with blocking buffer containing 10% donkey serum, 1% BSA, and 0.025% Triton X-100 for 45 min at room temperature. The sections were stained with purified anti-human/mouse CLA antibody at 1:100 dilution (Biolegend, 321302) at 4 °C overnight, followed by incubation in Biotinylated Goat anti-rat Ig secondary antibody (BD Pharmingen, 559286) for 60 min at room temperature. Further development was performed with Streptavidin-horseradish peroxidase (BD Pharmingen, 51-75477E) and DAB substrate kit (BD Pharmingen, 550880). Subsequently, the sections were counterstained with hematoxylin for 2 min.

### Immunofluorescence and microscopy

Tissue array were deparaffinised and incubated in Antigen Unmasking Solution (Vector laboratories, H-3300) at 98 °C for 20 min. Then the sections were treated with 0.3% Triton-X for 10 min at room temperature and were blocked with blocking buffer containing 5% donkey serum, 1% BSA, and 0.025% Triton X-100 for 60 min at room temperature. The sections were incubated with rabbit polyclonal anti-pSmad2/3 at 1:1,500 dilution (Cell Signalling Technology, 8828) and goat anti-amylase antibody (Santa Cruz, sc-12821) at 4 °C overnight, followed by incubation in Alexa fluor 647- conjugated donkey anti-mouse (Jackson ImmunoResearch, 715-605-150) and Alexa fluor 488-conjugated donkey anti-goat Ig secondary antibody (Jackson ImmunoResearch, 705-545-003) for 60 min at room temperature.

### cDNA preparation and qRT-PCR analyses

Total RNA was prepared using a Direct-zol RNA miniprep kit (Zymo Research), and used for cDNA synthesis using a high capacity cDNA reverse transcription kit (Applied Biosystems), according to the manufacturer’s protocol. Relative mRNA level was measured by qRT-PCR of each cDNA in duplicate with iTaq Universal SYBR Green Supermix (Bio-Rad) and the CFX96 Real-Time system (Bio-Rad). Normalizations across samples were performed using 18s RNA primers. Information regarding the primer and probe sets is available upon request.

### shRNA knockdown

Two validated SMAD4-specific shRNA lentiviral vectors and control shRNA lentiviral vectors were obtained from Sigma. Sorted primary human acinar cells were suspended in advanced DMEM/F-12 medium containing 2% serum. Cells were plated in 5% Matrigel-coated 96-wells plate (200,000/well) and incubated with shRNA expressing lentiviral particles for 24 hours. The cells were then cultured in serum-free media for an additional 24 hours. Cells were treated with TGF-β1 (5 ng/ml) for 72 hours beginning 48 hours after infection, with media changed daily, then analysed by flow cytometry.

### Statistical analysis

Data are presented as means ± s.e.m. P-values were acquired with the Student’s t or one-way ANOVA test using Prism (GraphPad Software), and P < 0.05 is considered statistically significant.

## Additional Information

**How to cite this article**: Liu, J. *et al*. TGF-β1 promotes acinar to ductal metaplasia of human pancreatic acinar cells. *Sci. Rep.* **6**, 30904; doi: 10.1038/srep30904 (2016).

## Supplementary Material

Supplementary Information
