# Supplementary material for: TGF-β1 promotes acinar to ductal metaplasia of human pancreatic acinar cells
Source: Sci Rep. 2016 Aug 3;6:30904. doi: 10.1038/srep30904 (PMC4971483; doi:10.1038/srep30904)
Supplement: Supplementary Information [file srep30904-s1.pdf]

Supplementary Information for

TGF-β1 promotes acinar to ductal metaplasia of human pancreatic acinar cells

Authors: Jun Liu, Naoki Akanuma, Chengyang Liu, Ali Naji, Glenn A. Halff , William K. Washburn, Luzhe Sun, and Pei Wang *

* To whom correspondence should be addressed. E-mail: wangp3@uthscsa.edu

This file includes:

Figure S1. Gating strategy and characterization of the primary human islet-depleted pancreatic fraction.

Figure S2. Identification of human ADM inducers in 3D culture.

Figure S1

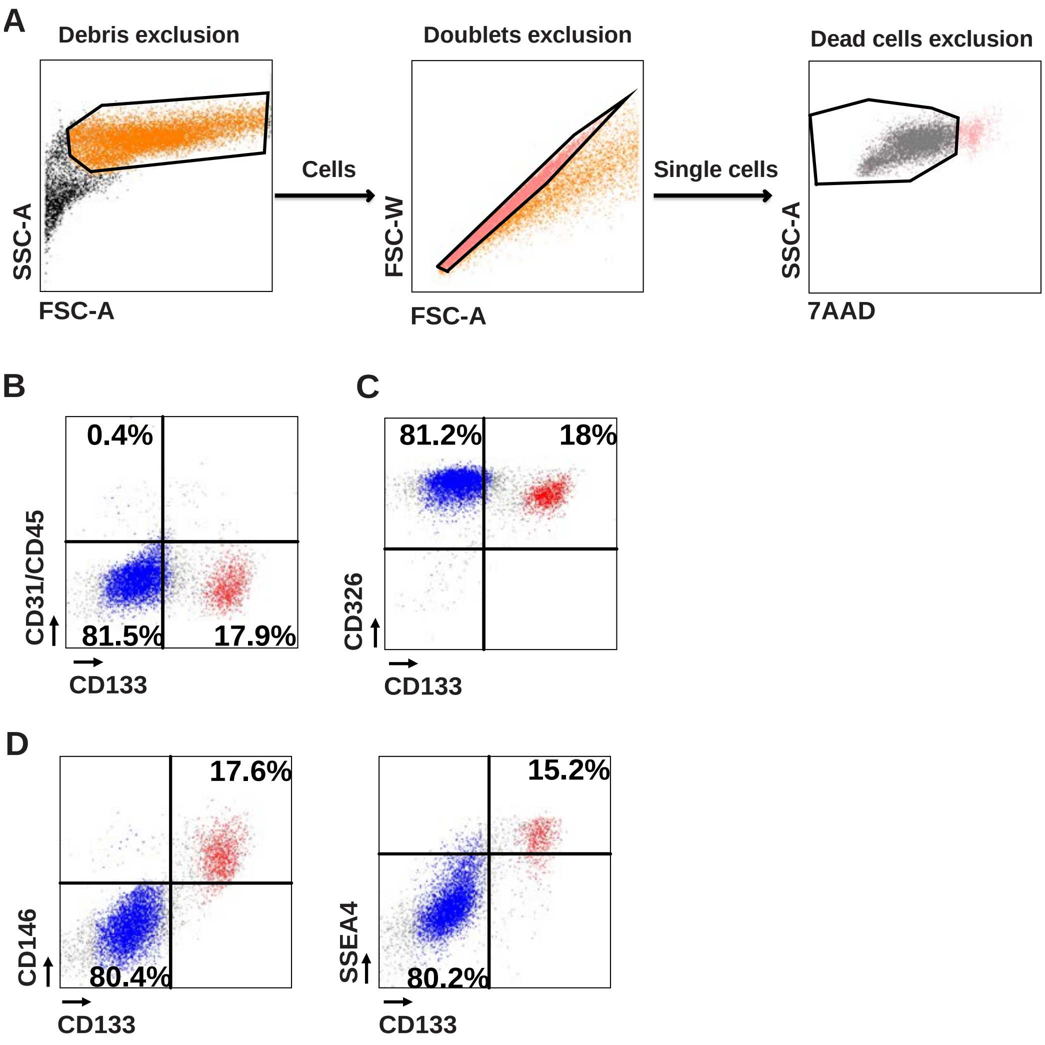

Gating strategy and characterization of the primary human islet-depleted pancreatic fraction. a) The panels indicate the exclusion of debris and doublets by FSC and SSC gating, and further exclusion of dead cells by 7AAD staining. b) A few cells are positive for CD31 or CD45. c) The majority of cells were $CD326^{+}$ epithelial cells. d) and e) CD146 and SSEA4 were preferentially expressed by ductal cells

Figure S2

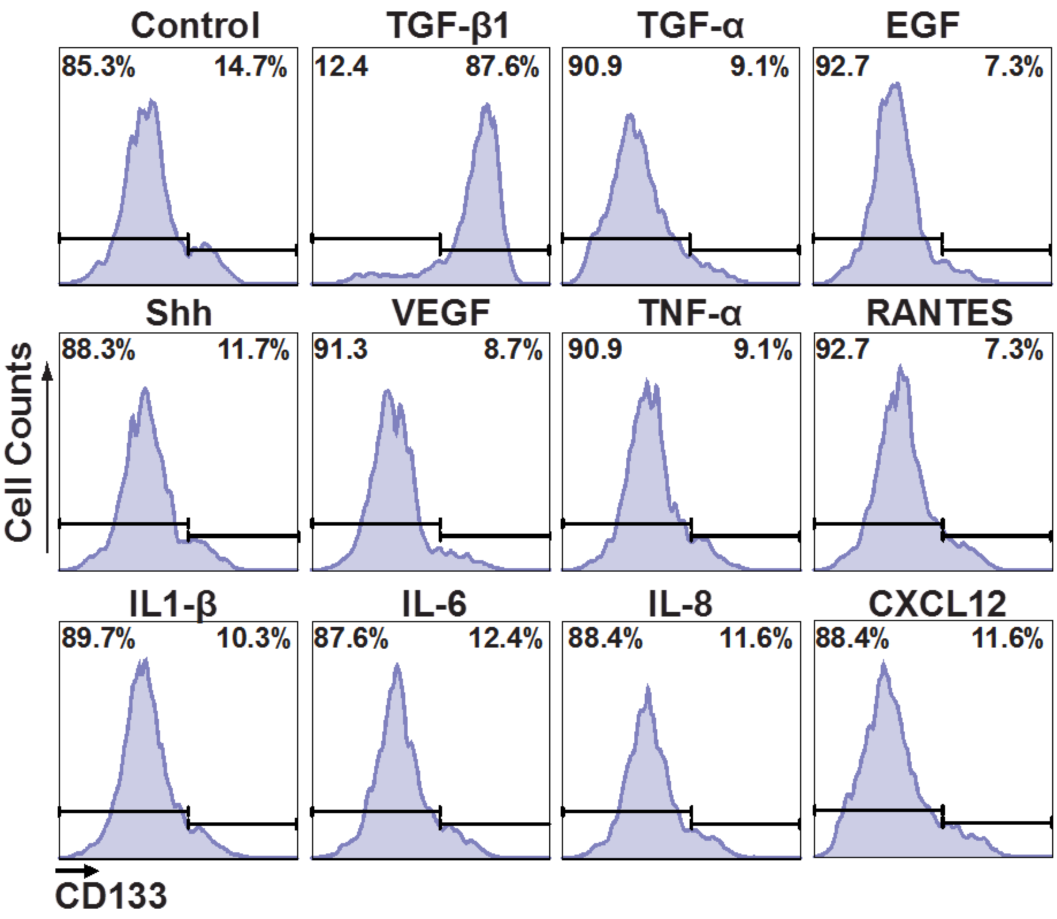

Identification of human ADM inducers in 3D culture. Screening murine ADM inducers for human primary pancreatic cells in 3D culture system. Fresh human pancreatic acinar cells were treated with the indicated cytokines and growth factors for 3 days. Flow cytometry analysis on UEA-1^{high} cells revealed that only TGF-β1 strongly induced CD133 expression in acinar cells, while other factors did not.
